# Hyperhomocysteinemia Is a Predictor for Poor Postoperative Angiogenesis in Adult Patients With Moyamoya Disease

**DOI:** 10.3389/fneur.2022.902474

**Published:** 2022-06-02

**Authors:** Qiheng He, Peicong Ge, Xun Ye, Xingju Liu, Jia Wang, Rong Wang, Yan Zhang, Dong Zhang, Jizong Zhao

**Affiliations:** ^1^Department of Neurosurgery, Beijing Tiantan Hospital, Capital Medical University, Beijing, China; ^2^China National Clinical Research Center for Neurological Diseases, Beijing, China

**Keywords:** moyamoya disease, angiogenesis, risk factor, homocysteine, hyperhomocysteinemia, prognosis

## Abstract

**Background and Purposes:**

The risk factors of poor postoperative angiogenesis in moyamoya disease (MMD) patients remain unknown. We aimed to investigate the association between hyperhomocysteinemia (HHcy) and postoperative angiogenesis of adult patients with MMD.

**Methods:**

A total of 138 adult patients with MMD were prospectively recruited from July 1 to December 31, 2019. After excluding 10 patients accepting conservative therapy and 77 individuals without postoperative digital subtraction angiography (DSA), all 51 MMD patients were enrolled, and 28 patients received bilateral operations separately. Patients were grouped according to postoperative angiogenesis and HHcy presentation, respectively. Clinical data and laboratory examinations were compared. Potential risk factors were evaluated by univariate and multivariate logistic regression analysis. Nomogram was further performed. The biological functions of homocysteine (Hcy) were explored *in vitro*.

**Results:**

Comparing to the normal, patients with poor postoperative angiogenesis were higher in serum Hcy (*p* = 0.004), HHcy ratio (*p* = 0.011), creatinine (Cr) (*p* < 0.001), uric acid (UA) (*p* = 0.036), Triglyceride (*p* = 0.001), high-density lipoprotein cholesterol (HDL-C) (*p* = 0.001), low-density lipoprotein cholesterol (LDL-C) (*p* = 0.009), ApoA (*p* = 0.022), apolipoprotein B (ApoB) (*p* = 0.013). Furthermore, HHcy was more common in men (*p* = 0.003) than women. Logistic analysis results showed that Hcy (*OR* = 0.817, 95% *CI* = 0.707–0.944, *p* = 0.006) was an independent risk factor. HHcy and Cr were significantly associated with poor postoperative angiogenesis in MMD patients. Further, Hcy could inhibit the proliferation, migration, and tube formation of human brain microvascular endothelial cells (HBMECs), which can be reversed by vascular endothelial growth factor (VEGF).

**Conclusion:**

The HHcy was significantly correlated with poor postoperative angiogenesis in adult patients with MMD. Hcy significantly inhibits HBMECs proliferation, migration, and tube formation. Furthermore, VEGF could reverse the inhibition effect induced by Hcy. Lowering the level of Hcy may be beneficial for postoperative MMD patients. Focusing on the pathophysiology and mechanism of HHcy might help to guide postoperative clinical management.

## Introduction

Moyamoya disease (MMD) is a rare cerebrovascular disease characterized by progressive stenosis of the intracranial internal carotid arteries (ICA) whose major branches with the emergence of co-existing compensatory abnormal net-like vessels (1–4). MMD is a major cause of stroke in children and young adults and has been observed in different ethnic backgrounds throughout the world, which is reported to be most common in Asian countries such as China, Japan, and Korea (5, 6). In surgical practice, indirect, direct, or combined revascularization is frequently applied, but the risk factors affecting poor postoperative angiogenesis need further research.

Homocysteine (Hcy) is reported to be a sulfur-containing amino acid, and an important intermediate in folate, vitamin B12, and one-carbon metabolism (7). It was reported that the genetic factors such as the mutation in 5,10-methylenetetrahydrofolate reductase (MTHFR) can change the plasma Hcy level. For normal and healthy individuals, the Hcy level in serum is between 5 and 15 μM, and an increase exceeding 16 μM is called hyperhomocysteinemia (HHcy) which may be harmful to vessels (8–10). Earlier studies have reported that the HHcy as an independent risk factor for poor health, such as cancer, coronary, Parkinson's disease, and Alzheimer's disease (8, 10–12). Recently, Ge et al. showed that the Hcy was associated with higher ischemic complications rates in MMD patients (3). However, the postoperative follow-up was limited, and the role of Hcy in postoperative angiogenesis remains unclear.

In our current study, the characteristics of adult MMD patients who underwent surgical options were collected to explore the relationship between Hcy and postoperative angiogenesis and performed experiments to explore the potential role of Hcy on brain vessels.

## Methods

### Study Participants

A total of 138 adult patients with MMD were prospectively recruited from July 1 to December 31, 2019 at the Department of Neurosurgery, Beijing Tiantan Hospital. A total of 10 individuals did not receive surgical treatment and 77 individuals without postoperative digital subtraction angiography (DSA) from the previous cohort were excluded. Finally, 51 MMD patients were prospectively enrolled in total, and 28 patients received bilateral operations separately. Guidelines of the Research Committee on Spontaneous Occlusion of the Circle of Willis in 2012 were used to diagnose MMD byDSA (13). All participants were signed the informed consent. The study was approved by the Ethics Committee of Beijing Tiantan Hospital, Capital Medical University (No. KY2016-048-01). The patients were grouped according to postoperative angiogenesis and HHcy presentation, respectively.

### Data Collection

The possible risk factors associated with poor postoperative angiogenesis were obtained, such as demographic data, clinical features, laboratory examinations, image examination, and surgical options. Age and sex are included in demographic information. Blood pressure, heart rate, and body mass index (BMI) were considered in clinical features. Laboratory examinations were levels of Hcy, blood glucose (Glu), albumin (ALB), total cholesterol (TC), triglyceride (TG), apolipoprotein A (ApoA), apolipoprotein B (ApoB), low-density lipoprotein cholesterol (LDL-C), high-density lipoprotein cholesterol (HDL-C), uric acid (UA), creatinine (Cr), white blood cells (WBC), and platelets (PLT). Suzuki stage was considered as imaging findings. Surgical options were summarized into direct revascularization or non-direct revascularization. After the participants had fasted for over 12 h in the morning, their blood samples were collected. The level of Hcy was extracted from medical records, and the plasma level of Hcy was determined by enzymatic cycling assay. An increase exceeding 15 μmol/L in serum Hcy level was diagnosed as HHcy. All patients accepted routine postoperative therapy and follow-up, including DSA 6 months postoperatively. The postoperative angiogenesis was evaluated independently by 2 senior physicians according to the Matsushima classification (14). In short, the surgical bypass covered the area including (A) more than two-thirds of the middle cerebral artery (MCA) distribution; (B) between two-thirds and one-third; and (C) only one cortical branch or no collateral circulation. Grade A and grade B were all considered to be good postoperative angiogenesis.

### Surgical Procedures

The 3 kinds of surgical procedures were used for MMD treatment, such as direct, indirect, and combined revascularization of the two (2). The detailed procedures refer to our previous work. In short, the direct bypass was the anastomosis of the cortical branch of the MCA and the superficial temporal artery (STA). In indirect bypass, the STA branch was isolated and placed on the cortical surface. Both direct and indirect bypass performed on the same hemisphere was called combined bypass.

### Statistical Analysis

The SPSS software (version 25.0) and R software (4.0.5) were used to perform the statistical analyses. The Pearson's chi-square test was used to compare the categorical variables. For continuous variables, the *t*-test and Mann–Whitney *U* test were utilized. The study employs logistic regression to investigate the independent factors. The 95% confidence intervals (*CIs*) and odds ratios (*ORs*) were calculated for potential risk factors related to poor postoperative angiogenesis. It was statistically significant when the *p* < 0.05 (two-sided).

## Results

### Characteristics and Laboratory Examinations of Postoperative Angiogenesis

A total of 79 subjects were analyzed in this study. The characteristics and laboratory examinations of postoperative angiogenesis were summarized in Table 1. A total of 37 (46.8%) subjects were in the poor postoperative angiogenesis group, such as 21 men and 16 women. The proportion of men was significantly higher in the poor postoperative angiogenesis group (56.8%) than in the good postoperative angiogenesis group (26.2%) (*p* = 0.006). In this cohort, the mean age was 40 ± 8 years. Between groups, subjects were significantly older in the poor postoperative angiogenesis group (*p* = 0.001). As for clinical features, BMI was significantly higher in the poor postoperative angiogenesis group (*p* = 0.005). There was no significant difference in the proportion of infarction as a primary symptom (*p* = 0.731), while it was 70.3% in the poor postoperative angiogenesis group and 66.7% in the good postoperative angiogenesis group (66.7%). In surgical options, 70.3% of subjects received indirect bypass in the poor postoperative angiogenesis group compared with 47.6% of subjects in the good postoperative angiogenesis group (*p* = 0.042). Those with poor postoperative angiogenesis showed a higher level of Hcy (*p* = 0.004), Cr (*p* < 0.001), UA (*p* = 0.036), TG (*p* = 0.001), HDL-C (*p* = 0.001), LDL-C (*p* = 0.009), ApoA (*p* = 0.022), and ApoB (*p* = 0.013). Meanwhile, the occurrence of HHcy in patients with poor postoperative angiogenesis was also significantly higher (*p* = 0.011).

**Table 1:** Baseline characteristics and laboratory examinations of postoperative angiogenesis.

**Variables**	**All patients (*n* = 79)**	**Postoperative angiogenesis**		***P*-value**
		**Poor (*n* = 37)**	**Good (n = 42)**	
Age, y, median (IQR)	37 (29–47)	40 (32–48)	34 (26–44)	0.01
Sex (%)				0.006
Male	32 (40.5)	21 (65.6)	11 (26.2)	
Female	47 (59.5)	16 (43.2)	31 (73.8)	
Primary symptom (%)				0.731
Infarction	54 (68.4)	26 (70.3)	28 (66.7)	
Non-infarction	25 (31.6)	11 (29.7)	14 (33.3)	
Medical history (%)				
Hypertension	10 (12.7)	6 (16.2)	4 (9.5)	0.372
Diabetes	6 (7.6)	2 (5.4)	4 (9.5)	0.491
Hyperlipidemia	0 (0)	0 (0)	0 (0)	*
Thyroid disease	0 (0)	0 (0)	0 (0)	*
Smoking	10 (12.7)	5 (13.5)	5 (11.9)	0.83
Drinking	4 (5.1)	2 (5.4)	2 (4.8)	0.896
Clinical feature, median (IQR)				
Heart rate, bpm	78 (74–80)	78 (72–80)	78 (75.5–80)	0.928
SBP, mmHg	127 (115–137)	127 (115–138)	122 (114.75–135)	0.297
DBP, mmHg	80 (75–90)	84 (76–94)	78 (74–90)	0.073
BMI, kg/m^2^	24.14 (22.64–26.83)	25.35 (23.26–27.68)	22.88 (20.63–25.84)	0.005
Surgical option (%)				0.042
Indirect bypass	46 (58.2)	26 (70.3)	20 (47.6)	
Non-indirect bypass	33 (41.8)	11 (29.7)	22 (52.4)	
Laboratory results, median (IQR)				
WBC count, 109/L	5.60 (5.10–6.60)	5.72 (5.18–6.64)	5.58 (5.08–6.46)	0.883
PLT, 109/L	229 (203–275)	232 (198–272)	228 (213–278)	0.680
Glucose, mmol/L	4.45 (4.18–4.8)	4.45 (4.03–4.87)	4.46 (4.18–4.80)	0.791
Creatinine, μmol/L	53.4 (45.3–64.1)	61.8 (48.85–71.50)	49.4 (43.50–56.98)	<0.001
Uric acid, μmol/L	288.3 (240.4–390.0)	318.8 (264.6–469.4)	281.7 (235.0–376.8)	0.036
Albumin, g/L	41.9 (40.0–44.5)	42.1 (39.8–43.9)	41.4 (40.0–44.9)	0.806
Triglyceride, mmol/L	1.35 (0.92–1.84)	1.78 (1.03–2.03)	1.14 (0.82–1.53)	0.001
Total cholesterol, mmol/L	4.09 (3.58–4.77)	4.24 (3.77–4.86)	3.88 (3.36–4.73)	0.116
HDL-C, mmol/L	1.08 (0.87–1.36)	0.94 (0.82–1.24)	1.23 (1.05–1.41)	0.001
LDL-C, mmol/L	2.58 (2.13–3.17)	2.81 (2.29–3.72)	2.21 (1.95–2.81)	0.009
ApoA, g/L	1.23 (1.07–1.43)	1.16 (1.00–1.26)	1.39 (1.10–1.47)	0.022
ApoB, g/L	0.84 (0.73–0.98)	0.89 (0.81–1.00)	0.77 (0.68–0.93)	0.013
Hcy, μmol/L	14.1 (11.26–16.41)	14.97 (12.01–17.60)	12.65 (8.77–14.68)	0.004
HHcy (%)	27 (34.2)	18 (48.6)	9 (21.4)	0.011
Suzuki stage (%)				0.165
I	1 (1.3)	0 (0)	1 (2.4)	
II	21 (26.6)	13 (35.1)	8 (19.0)	
III	41 (51.9)	16 (43.2)	25 (59.5)	
IV	11 (13.9)	7 (18.9)	4 (9.5)	
V	5 (6.3)	1 (2.7)	4 (9.5)	
VI	0 (0)	0 (0)	0 (0)	
PCA (%)	14 (17.7)	6 (16.2)	8 (19.0)	0.742

SBP, systolic blood pressure; DBP, diastolic blood pressure; BMI, body mass index; Hcy, homocysteine; HHcy, hyperhomocysteinemia; SD, standard deviation; IQR, interquartile range. Suzuki staging and posterior circulation involvement were defined on the operative side. ^*^P < 0.05, significant difference.

### Clinical Features of Patients According to HHcy

Clinical features of subjects according to HHcy are summarized in Table 2. The mean age in the HHcy group was significantly older than in the normal Hcy group (*p* = 0.046). Men in the HHcy group (63.0%) were also more than that in the normal Hcy group (28.8%), which was statistically significant (*p* = 0.003). As for primary symptoms, subjects with infarction as primary presentation in the HHcy group (88.9%) were significantly more than in the normal Hcy group (57.7%) (*p* = 0.005). In clinical features, the level of SBP, DBP, and BMI is statistically significant (*p* < 0.05). In laboratory examinations, the level of WBC (*p* = 0.003), CR (*p* = 0.013), UA (*p* = 0.004), HDL-C (*p* = 0.033), and TG (*p* = 0.006) in the poor postoperative angiogenesis group was significantly higher than in the good postoperative angiogenesis.

**Table 2:** Clinical characteristics of patients according to hyperhomocysteinemia (Hhcy).

**Variables**	**Hyperhomocysteinemia**		***P*-value**
	**Absent (*n* = 52)**	**Present (*n* = 27)**	
Age, y, mean (SD)	34 (28.3–44)	44 (32–47)	0.046
Sex (%)			0.003
Male	15 (28.8)	17 (63.0)	
Female	37 (71.2)	10 (37.0)	
Primary symptom (%)			0.005
Infarction	30 (57.7)	24 (88.9)	
Non-infarction	22 (42.3)	3 (11.1)	
Medical history (%)			
Hypertension	4 (7.7)	6 (22.2)	0.082
Diabetes	4 (7.7)	2 (7.4)	1
Hyperlipidemia	0 (0)	0 (0)	*
Thyroid disease	0 (0)	0 (0)	*
Smoking	6 (11.5)	4 (14.8)	0.728
Drinking	2 (3.8)	2 (7.4)	0.603
Clinical feature, mean (SD)			
Heart rate, bpm	78 (73–80)	80 (78–80)	0.135
SBP, mmHg	121 (114–135)	134 (118–140)	0.020
DBP, mmHg	78 (74–90)	90 (79–98)	0.025
BMI, kg/m^2^	23.19 (20.70–24.84)	26.67 (23.83–27.68)	0.001
Surgical option (%)			0.539
Indirect bypass	29 (55.8)	17 (63.0)	
Non-indirect bypass	23 (44.2)	10 (37.0)	
Laboratory results, median (IQR)			
WBC count, 109/L	5.45 (4.97–6.40)	6.22 (5.60–7.10)	0.003
PLT, 109/L	229 (212–277)	230 (173–272)	0.304
Glucose, mmol/L	4.47 (4.18–4.97)	4.4 (3.91–4.72)	0.363
Creatinine, μmol/L	51.15 (43.9–60.8)	57.2 (47.9–71.4)	0.013
Uric acid, μmol/L	275.5 (234.9–339.1)	332.1 (287.9–469.0)	0.004
Albumin, g/L	41.4 (39.5–44.1)	43.0 (40.7–45.1)	0.111
Triglyceride, mmol/L	1.21 (0.78–1.69)	1.66 (1.03–2.09)	0.006
Total cholesterol, mmol/L	3.91 (3.45–4.78)	4.22 (3.67–4.60)	0.984
HDL-C, mmol/L	1.22 (0.91–1.36)	0.94 (0.81–1.25)	0.033
LDL-C, mmol/L	2.49 (2.12–3.23)	2.66 (2.21–3.17)	0.788
ApoA, g/L	1.26 (1.10–1.47)	1.16 (1.00–1.35)	0.175
ApoB, g/L	0.85 (0.72–0.98)	0.84 (0.77–1.00)	0.291
Suzuki stage (%)			0.825
I	1 (1.9)	0 (0)	
II	15 (28.8)	6 (22.2)	
III	27 (51.9)	14 (51.9)	
IV	6 (11.5)	5 (18.5)	
V	3 (5.8)	2 (7.4)	
VI	0 (0)	0 (0)	
PCA (%)	8 (15.4)	6 (22.2)	0.450

SBP, systolic blood pressure; DBP, diastolic blood pressure; BMI, body mass index; Hcy, homocysteine; HHcy, hyperhomocysteinemia; SD, standard deviation; IQR, interquartile range. Suzuki staging and posterior circulation involvement were defined on the operative side. ^*^P < 0.05, significant difference.

### Logistic Analysis of Potentially Related Factors Associated With Poor Postoperative Angiogenesis

Potentially related factors for poor postoperative angiogenesis in adult MMD subjects were analyzed. The univariate analysis showed that age (*p* = 0.033), female (*p* = 0.007), BMI (*p* = 0.007), indirect bypass (*p* = 0.044), UA (*p* = 0.031), Cr (*p* = 0.001), TG (*p* = 0.003), HDL-C (*p* = 0.004), ApoA (*p* = 0.033), ApoB (*p* = 0.03), and Hcy (*p* = 0.016) were associated with poor postoperative angiogenesis in univariate logistic analysis (Table 3). After adjusting for all potential covariables, the results showed that creatinine (*OR* = 0.881, 95% *CI* = 0.783–0.992, *p* = 0.037) and Hcy (*OR* = 0.817, 95% *CI* = 0.707–0.944, *p* = 0.006) were independent factors related to the poor postoperative angiogenesis (Table 4). We also found that the HHcy was significantly associated with poor postoperative angiogenesis.

**Table 3:** Univariate analysis of risk factors for patients with poor postoperative angiogenesis.

**Variables**	**Univariate analysis**		
	**OR**	**95%CI**	***P*-value**
Age	0.948	0.902–0.996	0.033
Sex			
Female	3.699	1.435–9.532	0.007
Male	*	*	*
Primary symptom (%)			
Non-infarction	1.182	0.456–3.066	0.731
Infarction	*	*	*
Clinical feature, mean (SD)			
Heart rate, bpm	0.992	0.928–1.060	0.813
SBP, mmHg	0.975	0.943–1.008	0.143
DBP, mmHg	0.961	0.918–1.006	0.085
BMI, kg/m^2^	0.809	0.693–0.945	0.007
Surgical option (%)			
Indirect bypass	0.385	0.152–0.974	0.044
Non-indirect bypass	*	*	*
Suzuki stage	1.249	0.732–2.132	0.414
Laboratory results, median			
WBC count, 109/L	1.108	0.793–1.548	0.547
LY	0.992	0.429–2.292	0.984
PLT, 109/L	1.004	0.995–1.014	0.387
Glucose, mmol/L	0.825	0.542–1.255	0.368
Creatinine, μmol/L	0.927	0.887–0.969	0.001
Uric acid, μmol/L	0.995	0.991–1.000	0.031
Albumin, g/L	1.026	0.896–1.176	0.708
Triglyceride, mmol/L	0.281	0.122–0.674	0.003
Total cholesterol, mmol/L	0.765	0.440–1.332	0.344
HDL-C, mmol/L	16.142	2.468–105.592	0.004
LDL-C, mmol/L	0.553	0.286–1.068	0.078
ApoA, g/L	10.2	1.202–86.578	0.033
ApoB, g/L	0.047	0.003–0.747	0.03
Hcy	0.905	0.834–0.982	0.016
PCA	1.216	0.379–3.898	0.742

*SBP, systolic blood pressure; DBP, diastolic blood pressure; BMI, body mass index; Hcy, homocysteine; OR, odds ratio*.

*Suzuki staging and posterior circulation involvement were defined on the operative side*.

^*^P < 0.05, significant difference.

**Table 4:** Multivariate analysis on the risk of poor postoperative angiogenesis.

Variables^*^	**Multivariate analysis**		
	**OR**	**95%CI**	***P*-value**
Creatinine, μmol/L	0.881	0.783–0.992	0.037
Hcy	0.817	0.707–0.944	0.006

^*^Variables not finally entered into the model: age (P = 0.366), sex (P = 0.440), primary symptom (P = 0.186), BMI (P = 0.517), glucose (P = 0.069), uric acid (P = 0.142), albumin (P = 0.496), triglyceride (P = 0.164), total cholesterol (P = 0.214), HDL-c (P = 0.945), LDL-c (P = 0.147), ApoA (P = 0.758), ApoB (P = 0.779), WBC (P = 0.835), LY (P = 0.484), PLT (P = 0.403), surgical option (P = 0.156). BMI, body mass index; Hcy, homocysteine; OR, odds ratio. ^*^P < 0.05, significant difference.

### Nomogram

**Figure 1:**
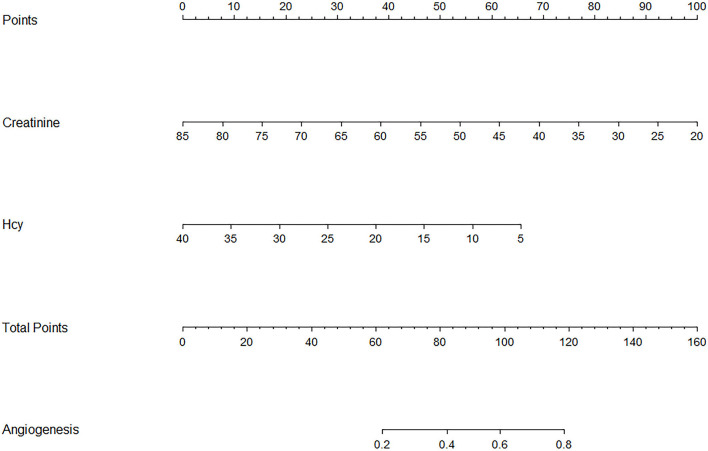
Nomogram of related factors influencing postoperative angiogenesis. Hcy represents homocysteine. Angiogenesis represents the possibility of good postoperative angiogenesis in adult moyamoya disease (MMD) patients.

To establish a predictive model of poor postoperative angiogenesis, we constructed a nomogram based on related factors in multivariate analysis, such as creatinine and Hcy. The nomogram achieved a c-index of 0.779, which reflects good predictive performance. The nomogram is shown in Figure 1. We also generated a calibration curve for the nomogram, which is shown in Supplementary Figure 1. The mean absolute error reached 0.019. Then, we performed the Hosmer–Lemeshow goodness-of-fit test, which indicated that the model was well calibrated (χ^2^ = 9.1299, *p* = 0.3315).

### Hcy Inhibits Proliferation, Migration, Tube Formation in HBMEC Which Is Reversed by VEGFA

**Figure 2:**
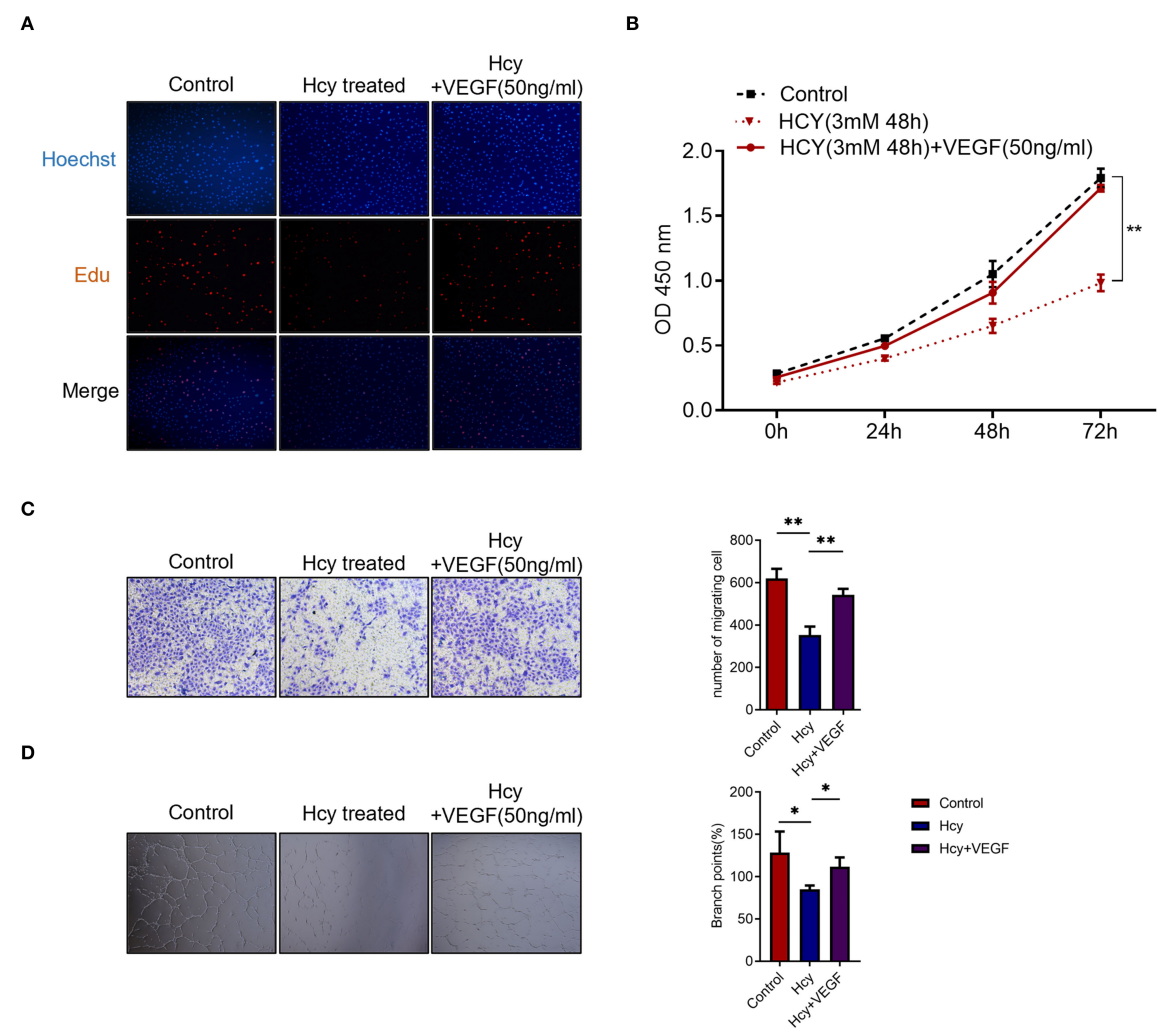
Homocysteine (Hcy) inhibits the HBMECs proliferation, migration, and tube formation, which is reversed by VEGF. **(A,B)** Edu assay and CCK-8 assay of HBMECs treated with Hcy and VEGF165. **(C,D)** Transwell migration assay and tube formation assay of HBMECs treated with Hcy and VEGF165. *p < 0.05, **p < 0.01.

To further study the biological function of Hcy in the angiogenesis of human cerebral vessels, we utilized human brain microvascular endothelial cells (HBMECs) to perform *in vitro* experiments. In the Edu assay, we found that the proliferation was significantly decreased by Hcy and was reversed by vascular endothelial growth factor (VEGF) treatment (Figure 2A). In the CCK-8 assay, we further confirmed that the proliferation rate was significantly decreased when treated with Hcy for 72 h (*p* < 0.01), and such effect could be reversed by VEGF (Figure 2B). Migration assay and tube formation assay were also performed to explore the influence of Hcy on vessel formation, and the results revealed HBMECs treated with Hcy were significantly decreased in migration (*p* < 0.01) and tube formation (Figures 2C,D). After being treated with VEGF, the effect of Hcy on HBMECs was reversed. The results indicated that Hcy inhibits proliferation, migration, and tube formation in HBMECs, and VFGF may become a potential treatment target for those patients with poor postoperative angiogenesis.

## Discussion

In this study, we prospectively enrolled adult MMD patients and investigated the potentially related factors of poor postoperative angiogenesis and found the increased level of Hcy (*p* = 0.004) and HHcy ratio (*p* = 0.011) were significantly associated with poor postoperative angiogenesis patients. It suggested that the Hcy plays a vital role in poor postoperative angiogenesis in MMD patients. We also established a nomogram and found patients with lower Hcy level is correlated with a better postoperative angiogenesis in MMD patients. Furthermore, we utilized HBMECs to conduct proliferation, migration, and tube formation assays and showed the inhibition effect of Hcy on cerebral angiogenesis can be reversed by VEGF.

The previous literature reported the link between Hcy and acute ischemic events, such as acute myocardial infarction (15). In this study, we revealed the potential effect of Hcy on long-term postoperative angiogenesis in adult MMD patients. Recently, HHcy was also reported to be associated with brain disorders, such as stroke (16). Our previous studies showed that the HHcy was associated with a higher risk of MMD and was correlated with postoperative acute ischemia within 7 days (3, 17). However, the relationship between poor cerebral postoperative angiogenesis and HHcy is not well-understood.

The Hcy, a key metabolite of methionine, is thought to participate in a variety of biological processes (18–20). Although how Hcy is involved in the pathogenesis of MMD is unclear, several possible mechanisms were reported in diseases. Recently, advances have shown that increased Hcy in serum level is a primary cause of cardiovascular diseases, diabetes, neurodegenerative diseases, and so on. Hcy was also involved in the initiation and progression of atherosclerosis by inhibiting the expression of miR-195-3p and in turn, enhancing the inflammation through IL-31 (21). In cardiovascular diseases, Hcy was reported to cause endothelial dysfunction through ENaC, or the toxicity related to iron containing proteins. Some studies reported that the Hcy could induce cell injury *via* Akt/eNOS pathway (22, 23). However, the possible role of Hcy on cerebral endothelial cells needs further research.

In MMD patients, the key mechanism leading to HHcy was thought to be associated with the mutation in MTHFR, which can interrupt the Hcy metabolism (24). And studies elucidated that Hcy may be involved in the pathogenesis of MMD by increasing MMP-9 in the vascular wall to induce inflammation (25, 26), but how Hcy is involved in postoperative cerebral angiogenesis remains unclear. Sato et al. reported that in STA–MCA bypass operations, HHcy is a risk factor for unsuccessful revascularization because it causes hypercoagulation (27). It also suggested that postoperative vitamin and folic acid replacement therapy contributes to an improved success rate of bypass surgery in patients with MMD, and postoperative replacement therapy may be beneficial in these patients (28–30). Recently, it was reported that Hcy can induce peripheral vessel apoptosis *in vitro* by modulating mitochondrial dysfunction, and autography *via* MIF/mTOR signaling (31–34). To study the effect of Hcy on postoperative cerebral angiogenesis, we conduct proliferation, migration, and tube formation experiments and found a significant decrease when HBMECs were treated with Hcy, and the effect of Hcy on HBMEC was further reversed by VEGF. The results confirmed that the Hcy can directly inhibit the brain angiogenesis to affect the long-term prognosis, and VEGF is a potential treatment for patients with poor postoperative angiogenesis.

In past studies on MMD, researchers focused on perioperative complications. However, studies on long-term follow-up and metabolism factors are limited. We utilized laboratory examinations to predict potential risk factors and further explore the mechanisms of Hcy on cerebral vessels. The logistic regression confirmed that the Hcy was an independent risk factor of poor postoperative angiogenesis. *In vitro* experiments confirmed the inhibition effect of Hcy on HBMECs. This indicated that postoperative angiogenesis can be worsened by HHcy in MMD patients. Interestingly, creatinine was also found to be significantly associated with the outcome of this study. Considering creatinine was at normal levels in whole patients enrolled in the study, this difference may be due to metabolites rather than pathological change, which may need further exploration. The results revealed the important biological role of HHcy on poor postoperative angiogenesis in MMD patients. For postoperative MMD patients, the level of Hcy should be monitored and well-controlled. Further, for patients with poor postoperative angiogenesis, HHcy potentially can be a therapeutic target and VEGF can be considered to be some kind of treatment. However, there were still limitations in our study. First, the sample size in the study was limited to a single-center study. Second, we did not include children MMD patients, which may be unaffected by HHcy due to the continuously high expression of PI3K/AKT pathway which can activate the cerebral vascular proliferation. Third, a follow-up up to several years is needed. Finally, the comprehensive mechanism and therapeutic drugs which may improve postoperative angiogenesis in MMD patients need further research.

## Conclusion

The study found that HHcy was significantly associated with poor postoperative angiogenesis in adult MMD patients. Hcy significantly inhibits HBMECs proliferation, migration, and tube formation. Furthermore, VEGF could reverse the inhibition effect induced by Hcy. Therefore, a new perspective that HHcy can act as a potential indicator and target is provided, and VEGF becomes a potential therapeutic drug to promote postoperative angiogenesis in MMD patients. Lowering the level of Hcy may be beneficial for postoperative MMD patients. In the future, focusing on the underlying mechanism of HHcy might help to guide postoperative clinical management.

## Data Availability Statement

The raw data supporting the conclusions of this article will be made available by the authors, without undue reservation.

## Ethics Statement

The studies involving human participants were reviewed and approved by Ethics Committee of Beijing Tiantan Hospital, Capital Medical University. The patients/participants provided their written informed consent to participate in this study.

## Author Contributions

XY, XL, and JW collected data. RW, YZ, and DZ supervised the data collection. QH analyzed the results, performed *in vitro* experiments, and wrote the manuscript. PG made the statistical comparison. PG and JZ designed the study. All authors contributed to the article and approved the submitted version.

## Funding

This study was supported by the National Key Technology Research and Development Program of the Ministry of Science and Technology of China (2015BAI12B04) and the National Natural Science Foundation of China (81701137 and 81870904).

## Conflict of Interest

The authors declare that the research was conducted in the absence of any commercial or financial relationships that could be construed as a potential conflict of interest.

## Publisher's Note

All claims expressed in this article are solely those of the authors and do not necessarily represent those of their affiliated organizations, or those of the publisher, the editors and the reviewers. Any product that may be evaluated in this article, or claim that may be made by its manufacturer, is not guaranteed or endorsed by the publisher.

## References

[1] Kuroda S Houkin K. Moyamoya disease: current concepts and future perspectives. Lancet Neurol. (2008) 7:1056–66. 10.1016/S1474-4422(08)70240-0 18940695

[2] Acker G Fekonja L Vajkoczy P. Surgical management of moyamoya disease. Stroke. (2018) 49:476–82. 10.1161/STROKEAHA.117.018563 29343587

[3] Ge P Zhang Q Ye X Liu X Deng X Wang J. Modifiable risk factors associated with moyamoya disease: a case-control study. Stroke. (2020) 51:2472–9. 10.1161/STROKEAHA.120.030027 32640948

[4] Mertens R Graupera M Gerhardt H Bersano A Tournier-Lasserve E Mensah MA. The genetic basis of moyamoya disease. Transl Stroke Res. (2022) 13:25–45. 10.1007/s12975-021-00940-2 34529262 PMC8766392

[5] Kleinloog R Regli L Rinkel GJ Klijn CJ. Regional differences in incidence and patient characteristics of moyamoya disease: a systematic review. J Neurol Neurosurg Psychiatry. (2012) 83:531–6. 10.1136/jnnp-2011-301387 22378916

[6] Kim T Oh CW Bang JS Kim JE Cho WS. Moyamoya disease: treatment and outcomes. J Stroke. (2016) 18:21–30. 10.5853/jos.2015.01739 26846757 PMC4747064

[7] Jakubowski H. Homocysteine modification in protein structure/function and human disease. Physiol Rev. (2019) 99:555–604. 10.1152/physrev.00003.2018 30427275

[8] Hasan T Arora R Bansal AK Bhattacharya R Sharma GS Singh LR. Disturbed homocysteine metabolism is associated with cancer. Exp Mol Med. (2019) 51:1–13. 10.1038/s12276-019-0216-4 30804341 PMC6389897

[9] Qin X Li Y Sun N Wang H Zhang Y Wang J. Elevated homocysteine concentrations decrease the antihypertensive effect of angiotensin-converting enzyme inhibitors in hypertensive patients. Arterioscler Thromb Vasc Biol. (2017) 37:166–72. 10.1161/ATVBAHA.116.308515 27834686

[10] Han L Wu Q Wang C Hao Y Zhao J Zhang L. Homocysteine, ischemic stroke, and coronary heart disease in hypertensive patients: a population-based, prospective cohort study. Stroke. (2015) 46:1777–86. 10.1161/STROKEAHA.115.009111 26038522

[11] Christine CW Auinger P Joslin A Yelpaala Y Green R Parkinson Study Group DI. Vitamin B12 and homocysteine levels predict different outcomes in early Parkinson's disease. Mov Disord. (2018) 33:762–70. 10.1002/mds.27301 29508904

[12] Elias MF. Reclaiming the importance of homocysteine as a marker of cardiovascular and neurologic disease. J Intern Med. (2021) 290:1098–9. 10.1111/joim.13305 34110042

[13] Research Committee on the P Treatment of Spontaneous Occlusion of the Circle of W Health Labour Sciences Research Grant for Research on Measures for Infractable D. Guidelines for diagnosis and treatment of moyamoya disease (spontaneous occlusion of the circle of willis). Neurol Med Chir. (2012) 52:245–66. 10.2176/nmc.52.245 22870528

[14] Matsushima T Inoue T Suzuki SO Fujii K Fukui M Hasuo K. Surgical treatment of moyamoya disease in pediatric patients–comparison between the results of indirect and direct revascularization procedures. Neurosurgery. (1992) 31:401–5. 10.1097/00006123-199209000-00003 1407421

[15] Borowczyk K Piechocka J Glowacki R Dhar I Midtun O Tell GS. Urinary excretion of homocysteine thiolactone and the risk of acute myocardial infarction in coronary artery disease patients: the wenbit trial. J Intern Med. (2019) 285:232–44. 10.1111/joim.12834 30193001 PMC6378604

[16] Maron BA Loscalzo J. The treatment of hyperhomocysteinemia. Annu Rev Med. (2009) 60:39–54. 10.1146/annurev.med.60.041807.123308 18729731 PMC2716415

[17] Li J Ge P Zhang Q Lin F Wang R Zhang Y. Hyperhomocysteinemia is a risk factor for postoperative ischemia in adult patients with moyamoya disease. Neurosurg Rev. (2021) 44:2913–21. 10.1007/s10143-021-01482-9 33506361

[18] Roth W Mohamadzadeh M. Vitamin B12 and gut-brain homeostasis in the pathophysiology of ischemic stroke. EBioMedicine. (2021) 73:103676. 10.1016/j.ebiom.2021.103676 34749301 PMC8586745

[19] Smith AD Refsum H. Homocysteine - from disease biomarker to disease prevention. J Intern Med. (2021) 290:826–54. 10.1111/joim.13279 33660358

[20] Murray LK Jadavji NM. The role of one-carbon metabolism and homocysteine in Parkinson's disease onset, pathology and mechanisms. Nutr Res Rev. (2019) 32:218–30. 10.1017/S0954422419000106 31303188

[21] Xiong J Ma F Ding N Xu L Ma S Yang A. Mir-195-3p alleviates homocysteine-mediated atherosclerosis by targeting Il-31 through its epigenetics modifications. Aging Cell. (2021) 20:e13485. 10.1111/acel.13485 34592792 PMC8520716

[22] Chen J Huang Y Hu X Bian X Nian S. Gastrodin prevents homocysteine-induced human umbilical vein endothelial cells injury via Pi3k/Akt/Enos and Nrf2/are pathway. J Cell Mol Med. (2021) 25:345–57. 10.1111/jcmm.16073 33320446 PMC7810955

[23] Borkowska A Ziolkowski W Kaczor K Herman-Antosiewicz A Knap N Wronska A. Homocysteine-induced decrease in huvec cells' resistance to oxidative stress is mediated by Akt-dependent changes in iron metabolism. Eur J Nutr. (2021) 60:1619–31. 10.1007/s00394-020-02360-8 32794021 PMC7987610

[24] Moll S Varga EA. Homocysteine and Mthfr mutations. Circulation. (2015) 132:e6–9. 10.1161/CIRCULATIONAHA.114.013311 26149435

[25] Korai M Kitazato KT Tada Y Miyamoto T Shimada K Matsushita N. Hyperhomocysteinemia Induced by excessive methionine intake promotes rupture of cerebral aneurysms in ovariectomized rats. J Neuroinflammation. (2016) 13:165. 10.1186/s12974-016-0634-3 27349749 PMC4924228

[26] Liu Y Song JH Hou XH Ma YH Shen XN Xu W. Elevated homocysteine as an independent risk for intracranial atherosclerotic stenosis. Aging. (2019) 11:3824–31. 10.18632/aging.102019 31188780 PMC6594800

[27] Sato K Morofuji Y Horie N Izumo T Anda T Matsuo T. Hyperhomocysteinemia causes severe intraoperative thrombotic tendency in superficial temporal artery-middle cerebral artery bypass. J Stroke Cerebrovasc Dis. (2020) 29:104633. 10.1016/j.jstrokecerebrovasdis.2019.104633 32122776

[28] Jacques PF Selhub J Bostom AG Wilson PW Rosenberg IH. The effect of folic acid fortification on plasma folate and total homocysteine concentrations. N Engl J Med. (1999) 340:1449–54. 10.1056/NEJM199905133401901 10320382

[29] Lee M Hong KS Chang SC Saver JL. Efficacy of homocysteine-lowering therapy with folic acid in stroke prevention: a meta-analysis. Stroke. (2010) 41:1205–12. 10.1161/STROKEAHA.109.573410 20413740 PMC2909661

[30] Wang X Qin X Demirtas H Li J Mao G Huo Y. Efficacy of folic acid supplementation in stroke prevention: a meta-analysis. Lancet. (2007) 369:1876–82. 10.1016/S0140-6736(07)60854-X 17544768

[31] Fan X Wang E He J Zhang L Zeng X Gui Y. Ligustrazine protects homocysteine-induced apoptosis in human umbilical vein endothelial cells by modulating mitochondrial dysfunction. J Cardiovasc Transl Res. (2019) 12:591–9. 10.1007/s12265-019-09900-6 31359360

[32] Wang X Wang Y Zhang L Zhang D Bai L Kong W. L-Cystathionine protects against homocysteine-induced mitochondria-dependent apoptosis of vascular endothelial cells. Oxid Med Cell Longev. (2019) 2019:1253289. 10.1155/2019/1253289 31885769 PMC6899331

[33] Zhang Z Wei C Zhou Y Yan T Wang Z Li W. Homocysteine induces apoptosis of human umbilical vein endothelial cells via mitochondrial dysfunction and endoplasmic reticulum stress. Oxid Med Cell Longev. (2017) 2017:5736506. 10.1155/2017/5736506 28630659 PMC5467318

[34] Zhang Y Zhang Y Tang J Zhao S Li C Huang YP. Oxymatrine inhibits homocysteine-mediated autophagy via mif/mtor signaling in human umbilical vein endothelial cells. Cell Physiol Biochem. (2018) 45:1893–903. 10.1159/000487912 29510402

