# Supplementary material for: Hyperhomocysteinemia Is a Predictor for Poor Postoperative Angiogenesis in Adult Patients With Moyamoya Disease
Source: Front Neurol. 2022 Jun 2;13:902474. doi: 10.3389/fneur.2022.902474 (PMC9201052; doi:10.3389/fneur.2022.902474)

Supplementary Figure 1. Calibration curve of the nomogram. Hosmer–Lemeshow is used to calibrate the nomogram, with a mean absolute error of 0.019.


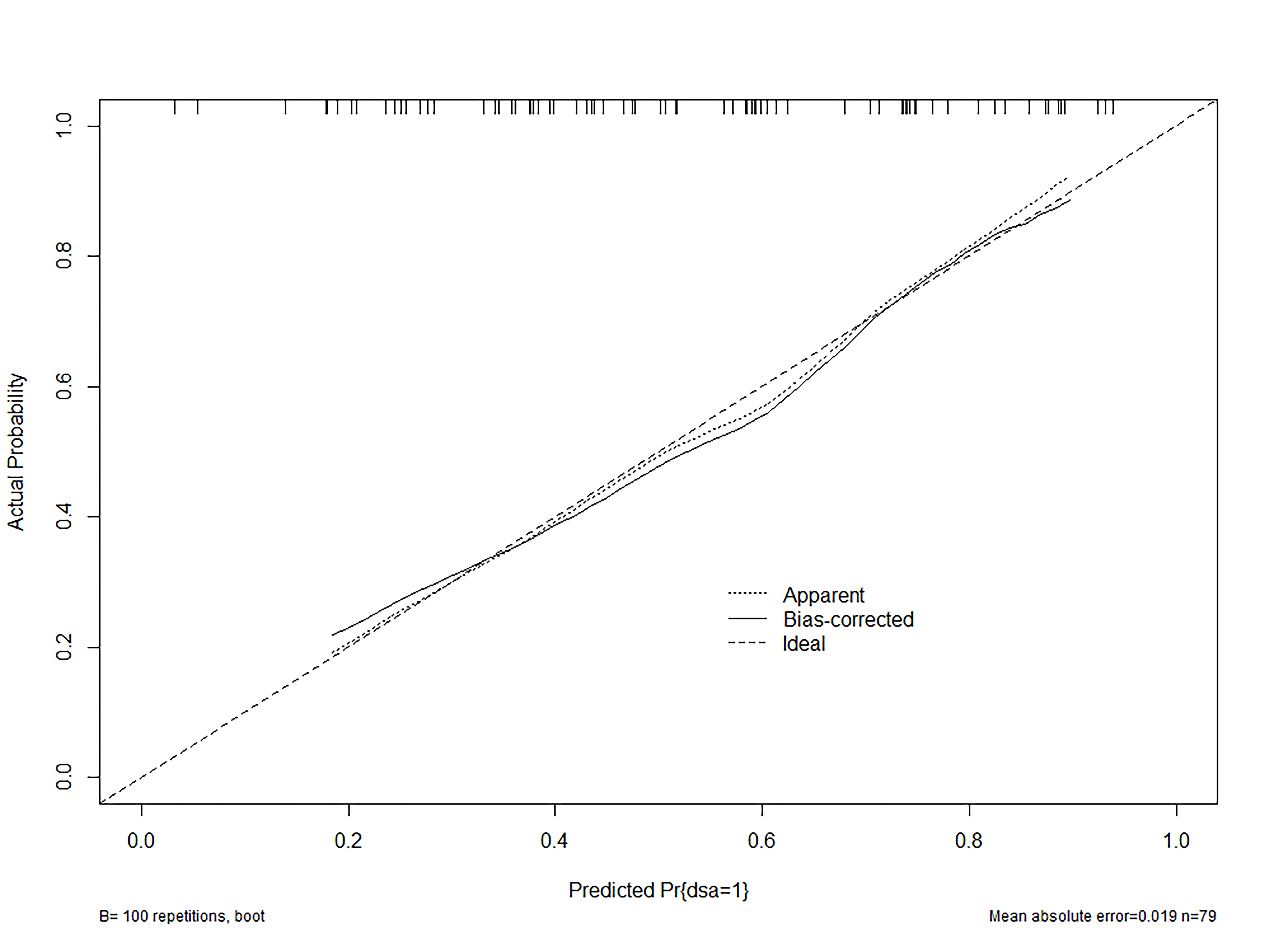

Supplement: Supplementary file 1 [file Table_1.DOCX]
